# Corticosteroid Regulation of Synaptic Plasticity in the Hippocampus

**DOI:** 10.1100/tsw.2010.48

**Published:** 2010-03-16

**Authors:** Nicola Maggio, Menahem Segal

**Affiliations:** ^1^ Department of Neurobiology, The Weizmann Institute of Science, Rehovot, Israel; ^2^ Talpiot Medical Leadership Program, Department of Neurology, The Chaim Sheba Medical Center, Tel HaShomer, Israel

**Keywords:** corticosterone, hippocampus, LTP, LTD, synaptic plasticity

## Abstract

Stress, via release of steroid hormones, has been shown to affect several cellular functions in the brain, including synaptic receptors and ion channels. As such, corticosteroids were reported to modulate plasticity, expressed as long-term changes in reactivity to afferent stimulation. The classical view of the effects of stress on synaptic plasticity and cognitive functions assumes an inverted U-shape curve, such that a low stress level facilitates and a high stress level (i.e., corticosterone levels) impairs cognitive functions. This universal view has been challenged recently in a series of studies that show that stress and corticosterone have immediate and opposite effects on the ability to express long-term potentiation (LTP) in the dorsal and ventral sectors of the hippocampus. This differential role of stress may be related to the different functions associated with these sectors of the hippocampus. Herein, we review the known effects of stress hormones on cellular functions and outline the role of molecular mechanisms in stress-related global functions of the hippocampus.

## INTRODUCTION

Stress has been defined as a real or perceived threat to an organism, which requires immediate compensatory responses for the maintenance of homeostasis[1]. Stressful stimuli cause release of the steroid hormone corticosterone (cortisol in humans) from the adrenal glands, which sets up the best response to the challenge by acting on corticosterone receptors, distributed throughout the body[2], with a particularly dense distribution in the brain[2]. Activation of the corticosterone receptors throughout the body recruits molecular cascades, which contribute to the ability of the organism to cope with stressful events. The cellular and molecular targets for the action of corticosterone include, in addition to basic metabolic processes, an effect on excitatory[22] and inhibitory[28] synaptic activity, as well as an effect on voltage-gated calcium channels (VGCC)[23,52] (Table 1).

The effects of corticosterone in the brain are mediated by activation of two types of receptors: the mineralocorticoid receptors (MR) and the glucocorticoid receptors (GR)[2,3,4]. Originally, it was suggested that both receptors act as nuclear transcription factors to modify protein synthesis and produce a slow,

**TABLE 1**
**Main Effects of Corticosteroids on Cellular Physiology of Limbic Cells**

| Brain Region | Receptor Type | Effect | Ref. |
|---|---|---|---|
| CA1 | iGR | ↑Amplitude L-type VGCC currents | [23,51,52] |
| | iGR | ↑Hyperpolarizing responses 5HT1A mediated | [52,53] |
| | mGR | ↑Amplitude GABAA-mediated currents | [28] |
| | mMR | ↑Frequency of mEPSCs | [8] |
| | mMR | ↓Frequency of mIPSCs | [28] |
| | mMR | Suppression of postsynaptic $K^+$ conductance $I_A$ | [55] |
| DG | iMR | ↑AMPA-mediated responses | [6,54] |
| BLA | iGR | ↑Excitability of pyramidal neurons | [56] |
| | iGR | ↓GABAA-mediated IPSPs | [56] |
| | mMR | ↑Frequency of mEPSCs | [58] |
| PVN | mGR | ↓Frequency of mEPSCs | [57] |

BLA, basolateral amygdala; PVN, paraventricular nucleus of the hypothalamus.

persistent change in the function of the cell[3,5,6]. More recent observations have reported the existence of a new family of membrane-bound MR and GR (mMR and mGR, respectively), which act through novel nongenomic pathways[7,8]. By this route, mMR and mGR can affect ionic conductances and thereby modify cell excitability and function[7,8]. Those membrane-bound receptors appear to differ from the intracellular receptors, not only in their location on the cell membrane, but also in their molecular structures[9], in their affinities for corticosterone, and finally in their mechanisms of action linked to G proteins[9] (Fig. 1).

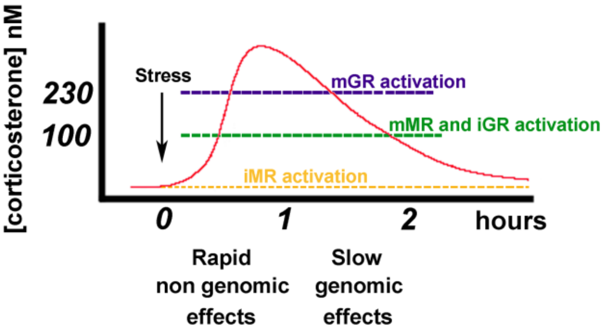

**FIGURE 1.** Time course of MR and GR activation following stressful stimuli. At a resting level, iMR are already saturated by the baseline levels of corticosterone. Rising concentration of corticosterone activates both mMR and iGR, whereas an additional increase in corticosterone levels also activates mGR. mMR- and mGR-mediated effects appear in a faster time course than those mediated by the intracellular receptors. Adapted from de Kloet et al.[7].

Intracellular MR (iMR) have a very high affinity for corticosterone and are highly expressed in all hippocampal subfields, as well as in cells of the central amygdala, lateral septum, and some motor nuclei in the brainstem[10]. Intracellular GR (iGR) have a relatively low affinity, are widely distributed throughout the brain, and are expressed both in neurons and in glial cells[10]. It has been proposed that iMR do not participate in the response to stressful stimuli, as they are already saturated by the low ambient levels of corticosterone at rest[4,10]. Conversely, iGR become gradually activated with rising levels of corticosterone following a stressful event[4,10]. In this respect, under physiological conditions, cells that coexpress both receptor types, such as principal cells in the CA1 region, the dentate gyrus, and the central amygdala, will shift between predominant iMR activation and concurrent iMR and iGR activation[11].

## STRESS AND PLASTICITY

The identification of the molecular cascades that govern the responses to corticosteroids in the brain shifted the attention to the involvement of corticosterone in neuronal plasticity, learning, and memory. In this respect, the effects of corticosterone on long-term potentiation (LTP) and long-term depression (LTD), the cellular mechanisms underlying learning and memory processes[12], began to unravel.

Initial studies indicated that induction of LTP in the hippocampal area CA1 is impaired in a rat exposed to behavioral stress, such as inescapable shock[13,14]. Administration of high doses of corticosterone either *in vivo*[15] or *in vitro*[16,17] produced the same effects, indicating that corticosterone is likely to mediate this action of stress. Specifically, corticosterone-induced impairment of LTP seems to be due to the activation of iGR, which depresses NMDA receptor–dependent LTP[18]. However, it was also shown that LTP can be enhanced in the presence of low to moderate concentrations of corticosterone, and LTP induction is impaired in absence of corticosterone[15]. These studies point toward a dose-dependent effect of corticosteroids on LTP induction, such that there is an inverted U-shaped relationship between the concentration of corticosterone and the ability to induce LTP[10,15].

Further studies have illuminated a more complicated picture of the effects of steroids on synaptic plasticity. Specifically, it seems that the same dosage of corticosterone that impairs NMDA-dependent LTP can indeed enhance VGCC-dependent LTP[18]. This kind of LTP is blocked by nifedipine, an L-type VGCC blocker[19]. It is thought to underlie the formation of fear memories in the amygdala[20,21] and can be evoked in the hippocampus as well[19]. Interestingly, in the hippocampus, corticosterone seems to enhance VGCC LTP through an iGR-dependent mechanism[18]. It has been proposed that this effect requires a genomic pathway, as it occurs after a long delay between the exposure to stress and/or corticosterone and the recordings[18], thus probably depending on the binding of GR homodimers to DNA that causes an increase in calcium currents[22,23].

Recent data from our group have shown that MR are also able to enhance VGCC LTP[24]. We have demonstrated that either stress or physiological concentrations of corticosterone can enhance LTP in the ventral hippocampus (VH), while inhibiting it in the dorsal hippocampus (DH)[24]. In particular, corticosterone enhances LTP through MR since a selective MR agonist, aldosterone, shares the same effect in the VH[24]. The proposed mechanism excludes an interaction between MR and NMDA receptors, as aldosterone by itself does not increase NMDA-dependent synaptic potentials[24]. Conversely, MR-induced LTP can be blocked by nifedipine, suggesting that VGCC are responsible for this effect[24]. It is likely that MR activate VGCC by modulating ionic conductances or changing the kinetics of VGCC activation. *In vivo* experiments have shown that MR activation is able to increase LTP in the DH as well[25]. Specifically, animals previously injected with a GR antagonist and then exposed to stress, such that only MR are activated, show a much larger LTP than controls. In contrast, those previously injected with an MR antagonist and then exposed to stress, such that only GR are activated, show a much lower LTP than controls[25]. These recordings were performed in the dentate gyrus (DG) and even though there are differences in the effects of stress and steroids between the DG and CA1[11], MR were still shown to mediate an enhancement of LTP.

These experiments raise several issues. It could be argued that as the experiments in the VH were conducted using an *in vitro* preparation, ambient corticosterone maintained normally through the circulation is washed out and, therefore, MR are not occupied and are ready to be activated by the superfused drug and enhance LTP in the VH. This does not reflect the situation in the intact animal, where the brain is constantly exposed to fluctuating concentrations of corticosterone. Specifically, MR should be already saturated by the resting concentration of corticosterone and should not respond to the stress-induced rise of corticosterone in the presence of a GR blockade, which is not the case[25]. Interestingly, even though both MR and GR are expressed in the VH, corticosterone action is mediated by activation of MR rather than GR. This reflects the observation that in the VH, MR concentration is double that of GR[26]. If so, MR should be saturated rapidly by the rising concentration of corticosterone and their effect should fade away faster in favor of the slower GR activation, which it does not. Thus, the simple, dose-dependent, inverted, U-shaped curve does not fully explain the modulatory functions of MR and GR on LTP in the different sectors of the hippocampus, necessitating the involvement of other factors.

A possible suggestion to clarify the MR-dependent enhancement of LTP can take into consideration the activation of mMR. It has been reported that those receptors act through a faster mechanism[7] and have lower affinities for corticosterone compared to their intracellular cognates[9]. Specifically, their affinity for corticosterone is equal to that of the iGR[9]. In addition, MR activation enhances LTP in the VH within 1 h, a short time window for genomic mechanisms to be activated[4,11], but seems compatible with the faster time course of the nongenomic routes (Fig. 1). Finally, mMR could be the preferential target for rising concentrations of corticosterone in the VH if one takes into account the similar affinities for corticosterone between mMR and iGR (Fig. 1), and the higher distribution of the former over the latter[26].

MR enhance LTP through VGCC activation. In our experiments, we could not detect any effect of iGR on VGCC LTP. This could most likely be due to the shorter time window of observation in our experiments compared to those done by others[18]. In any case, both MR and GR were reported to increase VGCC LTP[18,24,25]. This apparent contrast could probably be explained by considering the different time courses of MR and GR enhancement of VGCC LTP. Specifically, MR has an earlier effect than GR. In this sense, it could be that in the VH, stress mediates a quick enhancement of LTP by MR followed by a second, slow increase in LTP due to GR activation. This proposal is compatible with the proposed role of the VH as a key player in the pathway that conveys stressful information to the hypothalamus to organize the stress response[24,27].

## SYSTEM IMPLICATIONS

Following stress, the quick MR-mediated increase in LTP facilitates the flow of the stressful information from the VH to the ventral hypothalamus and other lower brain centers, so that the autonomic response to stress can be organized. Later on, the MR-mediated response fades away and the effect of GR dominates. As previously mentioned, GR enhancement of VGCC LTP has been shown to have a role in the formation of fear memories in the amygdala[20,21]. In this respect, GR could play the same function in the VH: the formation of the memory for the stressful event at the amygdala-VH pathway. Indeed, the evidence that MR and GR act on the same mechanism can have different purposes due to the time window of the respective outcomes that take place. Considering this, it could be interesting to study the relationship between the MR and GR responses in the VH. Specifically, more experiments are needed to determine the exact time schedule in the appearance of the GR response and its duration in comparison to that mediated by MR.

In the DH, the reduction of LTP is mediated by GR[24]. The exact pathway by which GR mediate this effect is yet to be addressed. However, as the case for MR in the VH, the effect of GR in the DH seems to occur in less than 1 h, a relatively quick response that is unlikely to be mediated by a genomic mechanism. GR could reduce NMDA-mediated LTP either by a direct or an indirect mechanism. As far as it concerns the indirect mechanism hypothesis, we have demonstrated that a GR agonist, dexamethasone, increases IPSC amplitude in the DH within minutes[28], consistent with the possible activation of mGR. Therefore, the increase in GABAA conductance could hyperpolarize the membrane preventing the cell from reaching the threshold of depolarization that unlocks NMDA receptors from the $Mg^{2+}$ block. All in all, our experiments show that GR affect LTP through a quick, probably nongenomic mechanism. Even though this hypothesis needs to be explored further, the fast suppression of LTP in the DH can underlie the switch in the weight between the DH and VH; by reducing DH LTP and simultaneously enhancing LTP in the VH, the stressful stimuli could temporarily suppress the cognitive route of the hippocampus to cortical structures and enable the transmission of the emotional information through the VH to the amygdala.

Conversely, LTD induction is facilitated by behavioral stress, through a mechanism that requires GR[29,30,31] and their effect on NMDA receptors[32,33]. However, in the hippocampus, we recently found that the effects of stress and corticosterone on LTD are indeed more complicated than originally thought. We replicated previous experiments by which both stress and corticosterone facilitate LTD through a GR-dependent mechanism in the DH, but we have also shown that LTD is impaired in the VH through a MR-dependent mechanism[34]. Specifically in the latter case, LTD is transformed in a slow-onset LTP following the exposure to stressful stimulation[34]. As is the case for LTP, changes in LTD either in the DH or VH were observed at approximately 1 h after the exposure to the stress, a time window that could be compatible with nongenomic mechanisms. The MR-induced conversion of LTD to LTP in the VH could be due to the activation of VGCC, which will further facilitate the ventral route to the amygdala.

Group I mGluR have been shown to enhance LTD in CA1[35,36], but, interestingly, they have been reported to induce a slow-onset potentiation in the DG[37]. In a previous study, we showed that, in the VH, application of DHPG, a group I mGluR agonist, increases the population spike amplitude in response to a baseline stimulation[38]. Taken together, these observations suggest that in the VH, a decrease in GABAergic inhibition can shift LTD to a slow-onset LTP through a group I mGluR-mediated mechanism (Fig. 2). This needs to be explored in further experiments.

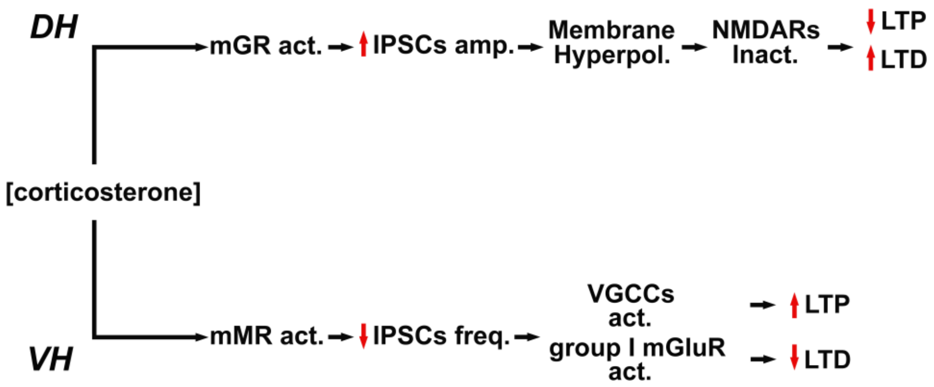

**FIGURE 2.** Proposed mechanism by which MR and GR differently regulate LTP and LTD in the DH and VH. In the DH, mGR activation increases IPSC amplitude. This results in a hyperpolarization of the pyramidal cell membrane and inactivation of NMDA receptors, therefore impairing LTP and enhancing LTD. In the VH, mMR activation reduces IPSC frequency. This determines an increase in the excitability of the pyramidal cells and raises the possibility of VGCC activation, thus enhancing LTP. In addition, a decrease in GABAergic inhibition can impair LTD through a group I mGluR-mediated mechanism.

## CONCLUSIONS

Corticosteroid regulation of synaptic plasticity in the hippocampus is affected by several factors. An inverted U-shape effect of corticosterone partially explains the observed modulation of LTP. Indeed, this hypothesis mainly refers to the activation of intracellular corticosteroid receptors and does not take into account the contribution of membrane-bound steroid receptors. In fact, mMR, which have a corticosterone affinity similar to that of iGR, will be activated at similar steroid concentrations. This implies that the effect of mMR appears earlier than that of iGR, thus inducing an enhancement of LTP instead of LTD. This might be the case in the VH.

An additional factor to be considered is the distribution of MR and GR in specific brain areas, and the ratio of membrane-bound vs. intracellular receptors expressed therein. This is because at the same affinity value for corticosterone concentration, the receptor that is highly expressed will lead the effects on synaptic plasticity.

The molecular structure of corticosterone receptors also seems to be important. MR, for example, exist in different molecular configurations[9], thus those receptors can be very different from one another. The diverse molecular structure could be linked to diverse intracellular pathways that differently influence neuronal functions.

Another issue that has to be considered is the clusters of brain areas that are involved in a particular stress situation. Various brain regions have specific properties and are incorporated into unique networks, so that even if corticosterone evokes the same effect at the single cell level, this would not always result in the same effect on LTP. For instance, both CA1 pyramidal neurons and granule cells in the DG highly express MR as well as GR[4,39]. In the DH, corticosterone and stress consistently suppress the induction of CA1 LTP *in vivo* and *in vitro*, unlike the case for the DG. High concentration of corticosteroid[40] or tail shocks[41] can indeed suppress LTP; however, in other situations, either no effect[42,43,44] or enhancement of LTP has been reported[45]. This is because LTP in the DG seems to be more dependent on indirect inputs from the amygdala[46,47,48].

Finally, the response to a stressor is also determined by the history of the organism. For instance, the induction of LTP is impaired in animals that have been exposed to repetitive stress in the weeks prior to the experiment, even if corticosterone levels, at the time of LTP induction, are compatible with the expression of a normal LTP[44,49]. Studies on the effect of maternal care on synaptic plasticity report that animals that received very little maternal care have poor LTP when they are adult, as opposed to animals that received high maternal care[50]. Interestingly, while LTP is suppressed by corticosterone in the latter group, it is enhanced in the former[50].

All in all, corticosteroid modulation of synaptic plasticity in the hippocampus seems to be more complex than previously thought and additional experiments are needed to address the role of membrane-bound as well as intracellular receptors on LTP/LTD regulation. In this respect, several genetic tools (RNAi, knock-out animals, etc.) are now available to better discern the responses of intracellular vs. membrane-bound receptors[59], independently from the time course of action of specific agonists. Their use should support the current pharmacological studies to address more specifically the contribution of each receptor in the regulation of synaptic plasticity.

## ACKNOWLEDGMENTS

This work was supported by a grant of the German/Israeli Research Foundation (DIP, I-1922) to M.S. N.M. is the recipient of a Talpiot Fellowship at Sheba Medical Center.
